# Arsenic toxicity to earthworms in soils of historical As mining sites: an assessment based on various endpoints and chemical extractions

**DOI:** 10.1007/s10653-023-01665-x

**Published:** 2023-06-27

**Authors:** Anna Karczewska, Iwona Gruss, Katarzyna Szopka, Agnieszka Dradrach, Jacek Twardowski, Kamila Twardowska

**Affiliations:** 1 Institute of Soil Science, Plant Nutrition and Environmental Protection, Wrocław University of Environmental and Life Sciences, ul. Grunwaldzka 53, 50-357 Wrocław, Poland; 2 Department of Plant Protection, Wrocław University of Environmental and Life Sciences, pl. Grunwaldzki 24a, 50-363 Wrocław, Poland; 3 Institute of Agroecology and Plant Production, Wrocław University of Environmental and Life Sciences, pl. Grunwaldzki 24a, 50-363 Wrocław, Poland

**Keywords:** *Eisenia fetida*, Soil, Arsenic, Toxicity, Bioavailability, Bioassay

## Abstract

*Eisenia fetida* is an earthworm species often used to assess the toxicity of contaminants in soils. Several studies indicated that its response can be unpredictable because it depends both on total concentrations of contaminants and also on their forms that differ in susceptibility to be released from soil solid phase. The issue is complex because two various uptake routes are concurrently involved, dermal and ingestion in guts, where the bioavailability of contaminants can considerably change. The aim of this study was to analyze the toxicity of arsenic (As) in various strongly contaminated meadow and forest soils, representative for former As mining and processing area, to earthworms *E. fetida* and its accumulation in their bodies. An attempt was made to find relationships between the response of earthworms and chemical extractability of As. In the bioassay, carried out according to the standard ISO protocol, different endpoints were applied: earthworm survival, fecundity measured by the numbers of juveniles and cocoons, earthworm weight and As accumulation in the bodies. The results proved that *E. fetida* can tolerate extremely high total As concentrations in soils, such as 8000 mg/kg, however, the individual endpoints were not correlated and showed different patterns. The most sensitive one was the number of juveniles. No particular soil factor was identified that would indicate an exceptionally high As susceptibility to the release from one of soils, however, we have demonstrated that the sum of non-specifically and specifically bound As (i.e. fractions F1 + F2 in sequential extraction according to Wenzel) could be a good chemical indicator of arsenic toxicity to soil invertebrates.

**Supplementary Information:**

The online version contains supplementary material available at 10.1007/s10653-023-01665-x.

## Introduction

Earthworms are invertebrates commonly occurring in soils of various habitats, in particular, in those rich in organic matter, typical for grasslands and forests (Curry, 2004; Karaca, 2010; Edwards et al., 2022). Their presence is of great importance for soil properties, as they improve soil structure by mixing the litter with the soil, and support the formation of soil aggregates and increase soil porosity (Blouin et al., 2013; Frazão et al., 2019 Lang et al., 2020). They contribute to organic matter decomposition and nutrient cycling (Edwards & Arancon, 2022; Medina-Sauza et al., 2019). Due to their widespread occurrence and wide range of ecological tolerance, earthworms have been shown useful indicators of soil contamination examined both in the field conditions and in the bioassays (Hirano & Tamae, 2011).

The tolerance of earthworms to adverse environmental factors, including their reactions to soil contamination, depends on many factors. Different earthworm species and ecotypes vary greatly in this respect (Zhang et al., 2018; Richardson et al., 2020). Several researchers described their induced, site specific tolerance (Langdon et al., 2003; Button et al., 2010; Huang et al., 2021). Their reactions under laboratory conditions can also be very different from those in the field (Giska et al., 2014; Liebeke et al., 2013; Palladini et al., 2022). The' responses of earthworms to soil contaminants, including arsenic, depend on the forms in which these contaminants occur in soils and on soil properties (Romero-Freire et al., 2015; Wang et al., 2016; Zeb et al., 2020; Xiao et al., 2022). Generally, earthworms are adversely affected by soil pollution with heavy metals and metalloids that can be taken up from soil by two possible routes: dermal and gut exposure (Arnold et al., 2003; Leveque et al., 2013; Garcia-Velasco et al., 2016). The main toxicity effect is oxidative stress (Wang & Cui, 2016; Yan et al., 2021).

*E. fetida* is a geopolitical earthworm species, easily accepting varied properties of the habitats if there is a constant access to bioavailable organic matter. It tolerates a wide range of temperatures and moisture levels (Dominguez & Edwards, 2004; Kostecka et al., 2022). It has been reported to prefer soils with a pH between 7.0 and 8.0, but it could also tolerate acidic pH, in the range 4.0–7.0 (Edwards & Arancon, 2022). *E. fetida* has been demonstrated as moderately sensitive to pollutants and showing the ability to inactivate and accumulate chemicals, like heavy metals, in their tissues (Hirano & Tamae, 2011). This is possible due to the species-specific detoxification system, which enables those organisms to tolerate higher contamination levels. It is not entirely clear to what extent the reactions of earthworms to soil contaminants depend on their easily soluble forms, particularly important for dermal exposure, and to what extent—on the forms that change in the earthworms' guts. Such changes in the mobility of metal(loids) in the gut can be caused by stimulation of soil microbial population, alteration of soil pH, presence of dissolved organic matter or the expression of metallothionein in the chloragogenous tissue surrounding the posterior alimentary canal (Garcia-Velasco et al., 2016; Langdon et al., 2005; Liebeke et al., 2013; Sizmur & Hodson, 2009). It is not clear, either, which of the properties of soil contaminants that the earthworms may alter in their guts are most important from the standpoint of toxicity (Sizmur & Hodson, 2009).

The sensitivity of *E. fetida* to a wide range of chemicals, and the fact that they can be easily cultured in the laboratory made this species, similarly as *E. andrei,* suitable for ecotoxicological bioassays (Bustos et al., 2015; Romero-Freire et al., 2015). Such bioassays play a very important role in the assessment of environmental risk caused by soil contamination, particularly when the pollution consists of various hazardous substances, and also when the factors that determine their bioavailability in given conditions are not fully known (Karczewska & Kabała, 2017). This is often the case of historical mining sites.

The early versions of standard bioassay with *E. fetida* proposed the use of artificial soils or natural non-contaminated soils spiked with solutions of toxic elements at increasing concentrations (ISO 11268, 1998; OECD 222, 2004), however, the later versions of the protocol (ISO 11268, 2012) also consider field collected contaminated soils. In the bioassays with *E. fetida*, different endpoints of the toxicity have been established. The most important are mortality (in the acute test), and inhibition of growth and reproduction as sublethal effects (Alves et al., 2013).

So far, quite a number of papers have been published on the reaction of various earthworm species, including *E. fetida*, to soil arsenic, but most of them dealt with soils spiked with soluble As, while a few studies concerned the soils collected from the field, especially those extremely strongly contaminated. More specifically, the impact of soil arsenic on earthworms was examined in the sites of a former arsenic mine at the Devon Great Consols, UK (Button et al., 2009, 2010, 2011), Deloro in Canada (Button et al., 2011), copper mines in Chile (Bustos et al., 2015), wood preservatives sites (Leduc et al., 2008), aged pesticide contaminated soils (Rahman et al., 2017), in the vicinity of the Shimen realgar mine, China (Yang et al., 2018) and gold mining activity in Korea (Lee et al., 2013). Of these studies, de facto only Bustos et al. (2015) and Lee et al. (2013) dealt with *E. fetida*, but in both cases arsenic was co-occurring with very high concentrations of various metals, and only in the latter case, the concentrations of As in soil samples were comparatively high to those reported in this paper. The high concentrations of other toxic elements in the soils examined in those studies did not allow to draw conclusions about the toxicity of arsenic alone.

In this study, arsenic was the main element present in soils in the concentrations considered toxic. The aim of the study was to examine the ecotoxicological response of earthworms *E. fetida* to extremely high concentrations of As in soils, taking into account various endpoints, and to analyze their relationships with soil properties and As extractability. The specific aim of the study was to check whether the reactions of earthworms to such high soil enrichment in As are correlated with As concentrations considered easily soluble or potentially bioavailable, determined in chemical extractions recommended as soil science standard procedures, and in the procedure specially dedicated to As, according to Wenzel et al. (2001). An additional aspect of the research was the comparison of the results obtained with *E. fetida* with those derived from other bioassays.

## Materials and methods

### Soil sampling sites

Soil material was collected from 6 representative sites situated in three historical arsenic mining centers: Złoty Stok, Radzimowice and Czarnów (Dradrach et al., 2020; Karczewska et al., 2013). Four of those sites (M1-M4) represented meadows that developed in the environments strongly polluted by arsenic mining and processing, and two others were located in the forests, directly on the mine dump (W1) or in the close vicinity of the Orchid dump (W2). The site M1 was a dry grassland where soils developed of pure tailings accumulated at the foreland of impoundment, while the site M2 was a typical hay meadow, occasionally flooded by tailings in the past. Collected soil material was sieved on site through a 5 mm screen to remove stones and coarser gravel fractions and then transported to the laboratory, air-dried, crushed and sieved to 2 mm prior to the experiment.

### Analyses of soil properties

Basic soil properties were determined in the aliquots of soil material, using the methods commonly applied in soil science (Tan, 2005). Soil texture was determined by a sieve and hydrometer method (Papuga et al., 2018). Chemical analyses were performed after soil grinding to a fine powder. Soil pH was measured potentiometrically in a suspension in 1 M KCl (1:2.5; v/v). Organic carbon (Corg) and nitrogen (N) were determined by a dry combustion method (Vario MacroCube, Elementar). Dissolved organic carbon DOC was measured in water extract (1:10; m/v). The content of available phosphorus (P avail.) was determined with the Egner lactate method (Kabała et al., 2018). “Pseudototal” concentrations of As in soils (further called “total”), were determined after microwave digestion with *aqua regia* (concentrated HNO_3_ + HCl, 1 + 3), following the standard procedure ISO 11466: 1995. Various chemical extractions were applied to determine the current and potential solubility of As and its and operationally defined bioavailability in soils. Potentially bioavailable and easily soluble As species were extracted from soils with standard methods, using 0.43 M HNO_3_, and 1 M NH_4_NO_3_ (1:2.5; m/v, 2 h), respectively, according to ISO 17586: 2016 and ISO 19730: 2008. Soil extraction with 0.43 M HNO_3_ was chosen as a simplified method for determination of potentially bioavailable or bioaccessible pool of metal(loid)s in contaminated soils, as suggested by various authors (Li et al., 2015; Rodrigues et al., 2018; Yao et al., 2021). Additionally, the As forms considered non-specifically and specifically sorbed, i.e. bound as outer-sphere and inner-sphere complexes, were determined in the sequential extraction according to Wenzel et al. (2001), as the sum of fractions F1 + F2. They were extracted with 0.05 M (NH_4_)_2_SO_4_ (1:25; m/v, 4 h) and 0.05 M (NH_4_)H_2_PO_4_ (1:25; m/v, 16 h) applied in a sequence to the same sample. The concentrations of elements, including arsenic, in the digests and extracts were measured by ICP-AES (iCAP 7400, Thermo Fisher Scientific). All these analyses were made in triplicates. The accuracy of total As measurement was checked with two reference materials: CNS 392 (trace elements in freshwater sediments, RTC, the Netherlands) and CRM 027 (trace elements in sandy loam 10, Fluka Analytical), certified for *aqua regia*-extracted elements, and the accuracy of As determination in various extracts was checked by standard addition.

### Effects of organic matter on the solubility of As in soils

A possible increase in As solubility that might have been caused by application of manure, that was used to feed the earthworms, was examined in simple incubation experiments. Soil samples were mixed with manure, applied at the rates 1:25 and 1:5, w/w, and incubated for 2 weeks at the moisture 70% of water holding capacity. After this time, soil extraction with 1MNH_4_NO_3_ was performed to determine the current solubility of As in soils, as described above.

Additionally, in a final discussion, the results of previous experiments were used, in which soils were incubated with two kinds of organic matter (manure and forest litter), applied at the rate 1:100. After the 2-week incubation, the concentrations of As in soil pore water were measured, and the ecotoxicity of pore water was determined in two bioassays: the Microtox that uses bacteria and Phytotox based on the inhibition of plant root elongation. The details were provided in earlier papers (Dradrach et al., 2019; Karczewska et al., 2022).

### Bioassays with *Eisenia fetida*

The bioassay with *E. fetida* was conducted according to the OECD standard 222 and ISO 11268-2: 2012, with some modifications. Earthworms were cultured in the peat and fed with unpolluted horse manure for three months before the beginning of the test. The juveniles were synchronized by transferring the cysts to the new culture containers. They were selected randomly and weighed individually before the test. Prior to weighing, they were washed with distilled water, and excess water was removed by placing the earthworms for a short period on a filter paper.

The bioassay was performed in two-liter vessels, each filled with 1 kg (dry weight equivalent) of soil. The soils were sterilized and moistened with distilled water 2 days before the test. Soil moisture was adjusted to 60 ± 5% of water holding capacities and maintained at that level during the test. The experiment was performed in 4 replicates for tested contaminated soils and 8 replicates for control. Twenty adult earthworms were selected, rinsed with water, weighed, and introduced into each test vessel. They were fed with horse manure spread on the soil surface at the beginning of the test and then once a week, each time at the rate 5 g d.w. per vessel. Covering the test vessels with organza material reduced evaporation. The bioassays were run for 8 weeks at the temperature 20 ± 2 °C and natural light. After that time, adult earthworms were counted, rinsed with water, weighed, and kept for 5 days on a moist filter paper in order to purify their guts. Then, they were humanely euthanized (by rapid freezing with liquid nitrogen), freeze-dried, and stored for analysis. The juveniles hatched from cocoons, as well as the cocoons, were separated from soils and counted.

Earthworm survival (calculated based on the number of earthworms that remained alive), the parameters of fecundity, i.e. the numbers of juveniles and cocoons, as well as the changes in earthworm mass, were used as the endpoints of bioassay.

### Arsenic accumulation by earthworms

An additional endpoint in this study was the concentration of As in the earthworm bodies. The freeze-dried earthworms were ground into a fine powder in a mortar. For determination of As in the biomass, the procedures commonly used for biological samples were applied. Briefly, 20 mg aliquots of each sample were pretreated overnight with 30% H_2_O_2_ and microwave digested in concentrated HNO_3_. The concentrations of As in the digests were measured by ICP–AES, as described above. Biological reference materials, relatively rich in As, i.e. BCR-414 (trace elements in plankton, BCR/JCR) and DC-73349 (trace elements in bush branches and leaves, CISRI/ NCS, China), were used for validation of analytical method.

### Data analysis and statistics

The endpoints of earthworm mortality and reproduction tests, as well as As concentrations in biomass, were compared to those obtained for the control soil using the analysis of variance (ANOVA) followed by Tukey post-hoc test. The Shapiro–Wilk test (*P* < 0.05) was performed to check the normality of data distributions. Pearson correlation coefficients were calculated to examine single relationships between the results of chemical analyses and ecotoxicological endpoints. Additionally, a principal component analysis (PCA) was performed in order to illustrate the distribution pattern of results and the associations between soil properties and bioassay endpoints. All statistical analysis were carried out using Statistica 13.0 software (StatSoft).

## Results and discussion

### Soil properties

The soils used in the experiment, devoid of skeletal parts, were relatively poor in the clay fraction (1–6%) and were classified in the textural classes of sands (S), loamy sands (LS) and sandy loams (SL). They contained relatively low amounts of Corg, in the range: 2.5–34.2 g/kg (Table 1). The M1 soil collected from a dry meadow at the foreland of tailings impoundment was the poorest in organic matter, while the highest concentration of Corg was found in the W2 forest soil.

**Table 1:** Sampling sites, soil properties and concentrations of potentially toxic metal(loid)s

Soil no.	Site description	Texture, percent of fraction			Textu-ral class	Corg g/kg	DOC mg/kg	Ng/kg	pH	CEC cmol+/kg	P avail. mg/kg	Total concentrations, mg/kg					
		Sand	Silt	Clay								As	Cu	Pb	Zn	Ni	Cd
C	Control, unpolluted soil	80	17	3	LS	16.8	0.10	1.18	6.05	13.8	64	2.4	8.9	11.2	38.1	9.7	0.65
M1	Foreland of tailings impoundment	76	21	3	LS	2.5	0.10	0.38	7.40*	20.2	194	8000	97.6	28.4	67.8	24.3	0.78
M2	Meadow on the Trująca floodplain, close to M1	53	41	6	SL	24.5	0.37	3.09	6.32	40.8	242	5020	56.3	282	790	33.9	1.50
M3	Meadow on the Trująca floodplain	77	22	1	LS	18.9	0.19	1.32	5.68	23.3	38	856	36.6	98.2	112	17.6	0.75
M4	Meadow in Czarnów	53	45	2	SL	16.5	0.13	0.98	4.40	18.6	7.1	235	26.4	38.6	118	20.7	1.02
W1	Radzimowice, mine dump close to Arnold adit	86	11	3	S	10.9	0.22	0.86	4.01	6.42	6.7	5170	206	370	278	14.5	0.93
W2	Złoty Stok, forest close to the Orchid Dump	55	44	1	SL	34.2	0.54	2.55	3.96	16.6	41	2024	62.8	102	153	30.8	1.31

*Soil M1 contained 2.1% CaCO_3_

Total concentrations of As in soils were generally very high, and ranged in a wide range: 235–8000 mg/kg. All those concentrations exceeded the permissible values, i.e. those considered unconditionally safe, set by Polish law at the levels 10–50 mg/kg for agricultural lands and 50 mg/kg for woodlands and forests. The exceedance of permissible values results in the requirement to carry out a risk analysis (Karczewska & Kabała, 2017). In a preliminary step of such unalysis, soil enrichment in As and other potentially toxic elements can be characterized by a geoaccumulation Index (Igeo). It was originally proposed by Müller (1981) for river sediments, and adapted by many researchers to soils (Kowalska et al., 2018). This index, of logarithmic character, allows for the assessment of soil contamination with particular element based on its concentration in top soil horizon and the value of geochemical background. Accordingly, the Igeo values < 1 are typical for uncontaminated sediments or soils, while Igeo > 3 indicates moderately or heavily contaminated soils, and Igeo > 5 denotes an extreme soil contamination. The concentrations of As in the soils examined in this study, assessed in the light of the Igeo, were classified as extreme pollution (Igeo > 5, in the range 5.1–10.1), whereas the concentrations of other potentially toxic elements were assessed as either low (unpolluted soils) or slightly elevated (moderately polluted), which corresponded to the Igeo < 3.3 (Table S1). The Igeo values confirmed that As was the main factor of toxicity in the soils under study.

A characteristic feature of forest soils (W1 and W2), important from the point of view of As solubility, was their strongly acidic reaction (pH 4.01 and 3.96, respectively), while the pH of meadow soils was in the range of 4.40–7.40. The M1 soil, with the highest pH, contained 2.1% of carbonates.

The forms of As soluble in 0.43 M HNO_3_, considered potentially bioavailable, accounted for a relatively large share in the total As pool in soils, in a fairly wide range 14–66% of total As (Table 2). The sum of forms F1 + F2 did not vary so widely, remaining in the range 13–17% of total As. The currently soluble form of As, extractable with 1 M NH_4_NO_3_ accounted for less than 0.5% of the total As, which corresponded to absolute concentrations in the range of 0.2–12.9 mg/kg. The incubation experiment with manure proved that the addition of manure caused a considerable increase, by several-fold, in the current solubility of As in soils (Table 2). The earlier studies also confirmed that application of organic matter, added to soil as manure or forest litter, resulted in a strong increase in As concentration in soil pore water, that in the case of M2 soil was the highest and reached 80.4 mg/L after a 2-day incubation (Table S2). This may be important when interpreting the results of bioassays with earthworms, in which manure was used to feed them.

**Table 2:** As extractability from untreated and manure-amended soils

Soil no.	Extractable As						1 M NH_4_NO_3_,—extractable As after treatment with manure at the rate	
	0.43 M HNO_3_		(F1 + F2)*		1 M NH_4_NO_3_		1:25 w/w	1:5 w/w
	mg/kg	% of total	mg/kg	% of total	mg/kg	% of total	mg/kg	mg/kg
C	0.5	21	0.15	6	< 0.01	< 0.4	0.02	0.02
M1	5310	66	393	5	12.9	0.16	31.4	57.2
M2	2720	54	860	17	7.32	0.15	20.1	29.1
M3	521	61	118	14	3.86	0.45	5.80	8.50
M4	47	20	24	10	0.29	0.12	0.08	0.39
W1	738	14	134	3	0.22	< 0.01	0.63	2.60
W2	977	48	278	14	1.91	0.09	1.11	11.4

*Non-specifically and specifically sorbed As—determined as the sum of fractions F1 + F2 in the sequential extraction acc. to Wenzel et al. (2001), extracted with 0.05 M (NH_4_)_2_SO_4_ and 0.05 M (NH_4_)H_2_PO_4_, applied in sequence

### Earthworm survival, weight change and fecundity

The analysis of experimental data, in particular the coefficients of variation and standard deviation for all endpoints obtained in the control soils, indicated that they were never above 30%, which means that the tests met the validity criteria recommended by the ISO guidelines. All endpoints, except of earthworm weight, and As concentrations in earthworm biomass strongly depended on soil properties and differed among the soils. In general, it was found that total As concentration in soil was not the crucial factor that determined the response of earthworms. Similar results were also reported by other researchers (Alves et al., 2018; Romero-Freire et al., 2015) who conducted the experiments on spiked soils and emphasized the importance of factors that determined arsenic solubility in soils, such as pH, soil texture and organic matter content. The effects of pH seem to be crucial mainly because soil pH strongly affects the solubility of As, while its direct impact on earthworms in not that important because *E. fetida* itself tolerates a broad range of soil pH from 4.0 to above 7.0 (Edwards & Arancon, 2022).

**Fig. 1:**
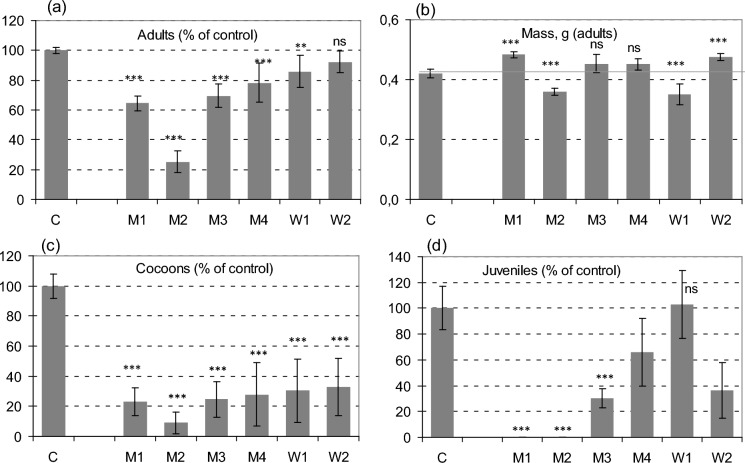
The results of bioassay with earthworms determined with various endpoints **a** the number of adults (as a measure of survival); **b** individual weight of adults; **c** fecundity measured by the number of cocoons, **d** fecundity measured by the number of juveniles. Error bar stand for 95% confidence intervals. Asterisks indicate significance of differences between the As-contaminated soils and control (C): * *P* < 0.05, ** *P* < 0.01, *** *P* < 0.001, ns—not significant

The results of earthworm survival differed considerably between the meadow soils and forest soils. In all the meadow soils M1-M4, earthworm survival significantly decreased compared to the control soil, while in the W1 and W2 forest soils only a statistically insignificant decrease in survival was reported (Fig. 1a). The percentage of surviving earthworms was the lowest (with the mean value of 25%) in the M2 soil, that represented the relatively fertile, moderately moist meadow in the Trujaca river valley, with total As concentration of 5200 mg/kg. This soil contained the highest concentration of non-specifically and specifically bound As (F1 + F2 fractions in sequential extraction by Wenzel et al., 2001), i.e. 860 mg/kg (Table 2). It was not explained why these As forms constituted such a large share in this particular soil. Undoubtedly, the mechanisms of As binding in this soil should be analyzed in more detail, including the mineralogical analysis of (hydroxy) iron oxides, the main soil sink of As, but this issue goes beyond the scope of the present paper. It should be added, however, that in the case of this soil, also the highest concentrations of As in the pore water were recorded, especially after the addition of manure (Table S2). This soil contained also a high concentration of As considered currently soluble, extracted with a 1 MNH_4_NO_3_, although the highest current solubility of As was reported from the M1 soil, in which it reached 12.9 and 57.2 mg/kg, without and with manure, respectively (Table 2).

The mean weight of the earthworms, determined after 8 weeks, did not considerably differ between those held in contaminated soils and those in the control (Fig. 1b). In the M2 and W1 soils, there was a small, buy statistically significant (*p* < 0.05) decrease in the average weight of adults, but in the other soils the effect was quite opposite, i.e. the earthworm weight increased. The latter effect should be probably explained by the fact that the weight was only determined for those individuals that survived, which may have had a particular individual resistance to arsenic. A similar interpretation was provided by Alves et al. (2018) who also reported a significant trend to increasing earthworm weights at the highest As concentrations in soils. They explained it by a higher tolerance of the biggest earthworms to arsenic exposure, or by the fact that those individuals that survived benefitted from reduced density in soil and higher food availability that enabled them to grow bigger. In the present study, however, there was not a negative correlation between earthworm survival and weight (Fig. 1a, b). Some researchers explained the reduction of earthworm growth by the high clay content of soils, in which *E fetida* became shrunken and stiff, or by low pH, below pH 5.5 (Lee et al., 2013), but it was not a case of the present study. There was no any significant dependence on pH either (Table S3).

The reproduction of earthworms was much more sensitive to As than their survival, and the patterns of two reproduction endpoints, i.e. the numbers of cocoons and juveniles, were different (Fig. 1c, d). In the M1 and M2 soils no juveniles were found at all, and the average number of cocoons in those soils, compared to the control, was 22 and 8%, respectively. In the other soils, the number of cocoons was also considerably low and did not exceed 33%. The number of juveniles was in almost all contaminated soils lower than that in the control, except for the W1, the highly acidic forest soil on the mine dump. It should be added that the number of juveniles showed the highest variability among the replicates, confirmed by the highest SD values, compared to the other endpoints. A similarly large variation in juvenile production was found by Romero-Freire et al. (2015), which the authors explained by the variation in soil properties. In the current experiment, significant negative correlations (*p* < 0.05) between the survival and reproduction endpoints (both the number of cocoons and juveniles) and the concentrations of “available” phosphorus in soil (Table S3) are also noteworthy, which can probably be explained by the chemical similarity of As and P, and similar conditions under which they exhibit high solubility and As also high toxicity.

### Accumulation of As in the earthworm biomass

**Fig. 2:**
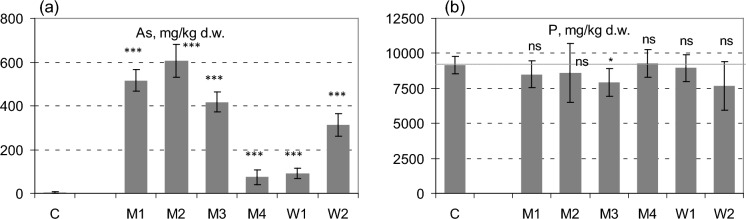
Concentrations of As (**a**) and P (**b**) in biomass of *E. fetida.* Error bar stand for 95% confidence intervals. Asterisk indicates the significance of differences between the As-contaminated soils and control (C): at *P* < 0.05; ns—not significant

The average concentration of As in the control earthworm biomass was 4.5 mg/kg d.w., which corresponds to the values reported by several other authors. In a review paper, Richardson et al. (2020) provided the values 4.0 and 5.3 mg/kg d.w., respectively, as the median and mean As concentrations from 56 studies. Wang et al. (2016) reported a similar value, 5.6 ± 1.1 mg/kg d.w., from the control soil. Accumulation of As by earthworms in the present experiment varied widely, and the average As concentrations in the biomass of worms held in contaminated soils were in the range 73–606 mg/kg d.w. (Fig. 2). The highest value was reported from the M2 soil, i.e. a meadow in the valley of the Trująca river, with the highest share of non-specifically and specifically bound As (F1 + F2) and the highest concentrations of As in pore water after a 2-week incubation both with and without manure (Table S2). The maximum As concentration in earthworm biomass, which in the present study was 606 ± 77 mg/kg, remains lower than the values reported by several other researchers. Fischer and Koszorus (1992) found considerable bioconcentration of As (up to 902 mg/kg d.w.) in *E. fetida* exposed to sublethal concentrations of As (10–87 mg/kg) in arsenate spiked soils. Maximal accumulation capacity obtained in the study by Romero-Freire et al. (2015) was 1019 ± 167 mg/kg d.w.; while at higher body concentrations, all earthworms died.

**Fig. 3:**
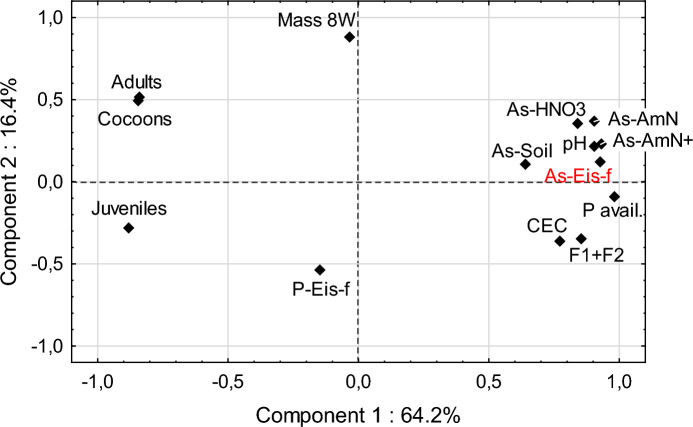
The PCA graph illustrating the relationships between the absolute concentrations and extractable forms of As in soils, soil properties, As and P concentrations in earthworm biomass (As-Eis-f and P-Eis-f), and the other endpoints of bioassay: mean biomass of adult earthworm after 8 weeks (Mass-8W), and the numbers of adults, cocoons and juveniles. As-Soil = total As concentration in soil, As-HNO3 and As-AmN—soil concentrations of As extractable with 0.43 MHNO3 and 1 M NH_4_NO_3,_ respectively, F1 + F2—As extractable in fractions F1 + F2 in sequential extraction acc to Wenzel et al. (2001). The abbreviations of soil parameters: as in the text

In the current study, the concentrations of As in earthworm biomass were significantly (*p* < 0.05) positively correlated with soil pH and 1 M NH_4_NO_3_-extracted As (Fig. 3), similarly as was the number of juveniles (Table S3). Similar correlations were reported by Lee et al. (2013). A correlation of As accumulation in earthworm bodies on the sum of F1 + F2 fractions in the extraction according to Wenzel, although positive, did not turn out to be statistically significant at *p* < 0.05, which may indicate that in the case of soils containing very high concentrations of As, the results of this extraction may overestimate As bioavailability. On the other hand, the concentrations of As in biomass were positively correlated with soil bioavailable phosphorus, which seems to result from similar solubility patterns of arsenic and phosphorus. It should be stressed, however, that the interactions of the ions of these elements are often difficult to predict. Several authors reported the competition between them and reduction of As uptake by various biota, including earthworms, in the presence of high concentrations of soluble phosphates (Anawar et al., 2018; Lee & Kim, 2008; Lewińska & Karczewska, 2013). Such a relationship was not observed in the present study, most likely because the accumulation of P in earthworm biomass was many times higher than that of As and did not vary significantly (Fig. 2).

### Comparison of various endpoints and As accumulation in earthworm biomass

Comparing the survival of *E. fetida* with other endpoints indicates that their patterns were different and poorly related to each other. The endpoint based on earthworm weight was the least sensitive (Fig. 1b), while the reproduction tests, with the number of cocoons and juveniles as endpoints, turned out to be the most sensitive. Greater sensitivity of reproduction tests than survival was also reported by Romero-Freire et al. (2015) in As spiked soils. The lack of correlation between two reproduction endpoints (cocoons and juveniles) was confirmed by PCA analysis (Fig. 3) and related single correlation coefficients (Table S3). Both of these endpoints showed a similar dependence on a principal component P1, but they depended differently on the P2 component.

**Fig. 4:**
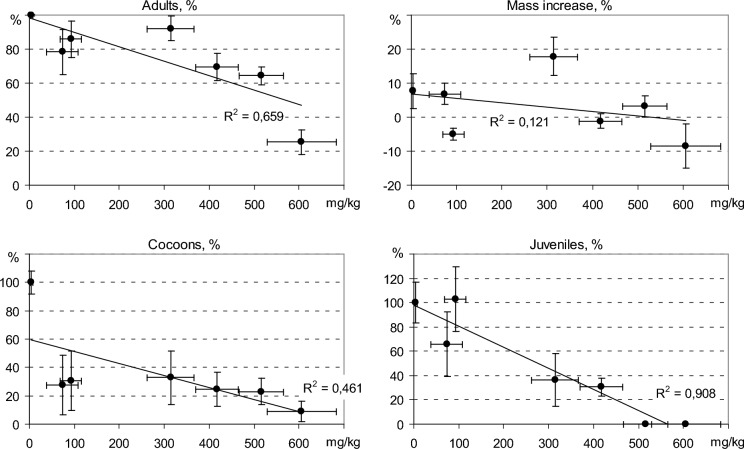
Dependence of the results of earthworm growth and reproduction versus As concentration in biomass. On the horizontal axis of all graphs: concentration of As in biomass, mg/kg d.w., on the vertical axes relative endpoint values, compared to the control soil. The figure shows the mean values and SD for individual soils

Two very strongly correlated endpoints (*p* < 0.001) were earthworm survival and the number of cocoons (Table S3), which is also shown by the PCA graph (Fig. 3). There was, however a factor that had the opposite effects on the number of cocoons and the number of viable juveniles. Based on single correlation coefficients (Table S3), it can be assumed that such a factor of significant importance was soil reaction pH. The number of juveniles was negatively correlated with the pH value and with the actual solubility of As in soil (i.e. 1 M NH_4_NO_3_-extractable As). This means that the development of juveniles was most severely limited under conditions of relatively high pH and high concentration of currently soluble As. The number of juveniles was the only earthworm endpoint that significantly correlated (*R* = − 0.935, *p* < 0.01) with the amount of As accumulated in earthworm biomass (Table S3). The other endpoints showed practically no relationship with the biomass concentration of As, which is illustrated in Fig. 4. Even at As in biomass below 100 mg/kg d.w., production of coccoons fell below 50% of that in control, which is in line with the findings by Bustos et al. (2015) who stated that the concentrations of As in the tissues of adult earthworms explained 45% of the variance of their cocoon production.

It is worth adding that the most sensitive endpoints of this study, i.e. the number of juveniles and As concentration in biomass, showed a good correlation with the endpoints of two other bioassays: a bacterial one (Microtox) and that based on the reduction of *Sinapis alba* root elongation (Phytotox) (Fig. 3, Tables S2 and S3) when carried out under the conditions conducive to the release of As i.e. with an addition of organic matter (Dradrach et al., 2019). This could suggest that the process of accumulation by earthworms involves the mechanism of As desorption from the solid phase of soils under the influence of organic substances, either exogenous, introduced for feeding, or endogenous, secreted by the earthworms themselves. The exceptionally high sensitivity of juvenile individuals to the toxic effects of As is interesting, which may be explained by the fact that the mechanism of inducing the expression of metallothioneins capable of detoxifying As, described by Langdon et al. (2005) for adults *Lumbricus rubellus*, is not yet operational in the juvenile *E. fetida.* This issue seems very interesting but would require more detailed research using the methodology used in invertebrate physiology.

## Conclusions

This study showed that *E. fetida* earthworms can tolerate extremely high levels of As in soils and that even a concentration as high as 8000 mg/kg, present in soil M1, reduced the survival by less than 40%. The research showed that the endpoints of bioassay with earthworms were not correlated with each other and showed different patterns, with the number of juveniles being the most sensitive, while there were no adverse effects of As on the individual weight of surviving earthworms. Particularly worth emphasizing is the fact that the highest toxicity and the strongest accumulation of As in the biomass of earthworms were found in the case of M2 soil, with the highest concentrations of non-specifically and specifically bound As (the sum of fractions F1 + F2) and the highest concentrations of As in soil pore water. However, any single factor that caused the exceptionally high susceptibility to the release and high toxicity of As in this soil has not been identified. These observations suggest that the sum of non-specifically and specifically bound As could be a very good indicator of toxicity of soil As to earthworms, in particular, in highly enriched soils. This hypothesis should be tested on a larger number of soil materials with different properties, contaminated with As of different origin and in different forms. Our study confirms that the reactions of earthworms to As in extremely enriched soils do not depend on total concentrations of this potentially toxic element or on the concentrations of its strongly bound forms, soluble in strong extractants, such as 0.43 M nitric acid, but on relatively weaker bound forms, extractable with weaker extractants, such as 1 M NH_4_NO_3_ or those used for F1 + F2 fractions in the sequential extraction according to Wenzel.

## Supplementary Information

Below is the link to the electronic supplementary material.

Supplementary file1 (DOC 100 KB)

## Data Availability

The data that support the findings of this study are available on request from Prof. Anna Karczewska.

## References

[CR1] Alves PRL, Cardoso EJ, Martines AM, Sousa JP, Pasini A (2013). Earthworm ecotoxicological assessments of pesticides used to treat seeds under tropical conditions. Chemosphere.

[CR2] Alves PRL, da Silva EB, Cardoso EJBN, Alleoni LRF (2018). Ecotoxicological impact of arsenic on earthworms and collembolans as affected by attributes of a highly weathered tropical soil. Environmental Science and Pollution Research.

[CR3] Anawar HM, Rengel Z, Damon P, Tibbett M (2018). Arsenic-phosphorus interactions in the soil-plant-microbe system: Dynamics of uptake, suppression and toxicity to plants. Environmental Pollution.

[CR4] Arnold RE, Hodson ME, Black S, Davies NA (2003). The influence of mineral solubility and soil solution concentration on the toxicity of copper to *Eisenia fetida* Savigny. Pedobiologia.

[CR5] Blouin M, Hodson ME, Delgado EA, Baker G, Brussaard L, Butt KR, Dai J, Dendooven L, Peres G, Tondoh JE, Cluzeau D, Brun JJ (2013). A review of earthworm impact on soil function and ecosystem services. European Journal of Soil Science.

[CR6] Bustos V, Mondaca P, Verdejo J, Sauvé S, Gaete H, Celis-Diez JL, Neaman A (2015). Thresholds of arsenic toxicity to *Eisenia fetida* in field-collected agricultural soils exposed to copper mining activities in Chile. Ecotoxicology and Environmental Safety.

[CR7] Button M, Watts MJ, Cave MR, Harrington CF, Jenkin GT (2009). Earthworms and in vitro physiologically-based extraction tests: Complementary tools for a holistic approach towards understanding risk at arsenic-contaminated sites. Environmental Geochemistry and Health.

[CR8] Button M, Jenkin GR, Bowman KJ, Harrington CF, Brewer TS, Jones GD, Watts MJ (2010). DNA damage in earthworms from highly contaminated soils: Assessing resistance to arsenic toxicity by use of the Comet assay. Mutation Research/genetic Toxicology and Environmental Mutagenesis.

[CR9] Button M, Moriarty MM, Watts MJ, Zhang J, Koch I, Reimer KJ (2011). Arsenic speciation in field-collected and laboratory-exposed earthworms *Lumbricus terrestris*. Chemosphere.

[CR10] Curry J, Edwards CA (2004). Factors affecting the abundance of earthworms in soils. Earthworm ecology.

[CR11] Dominguez J, Edwards CA, Shakir Hanna H, Mikhall WZA (2004). Vermicomposting organic wastes: A review. Soil zoology for sustain development in the 21st century.

[CR12] Dradrach A, Szopka K, Karczewska A (2019). Ecotoxicity of soil pore water on historical arsenic mine dumps—the effects of forest litter. Ecotoxicology and Environmental Safety.

[CR13] Dradrach A, Karczewska A, Szopka K, Lewińska K (2020). Accumulation of arsenic by plants growing in the sites strongly contaminated by historical mining in the Sudetes region of Poland. International Journal of Environmental Research and Public Health.

[CR14] Edwards CA, Arancon NQ (2022). The influence of environmental factors on earthworms. Biology and ecology of earthworms.

[CR15] Edwards CA, Arancon NQ, Bohlen PJ, Hendrix P (2022). Biology and ecology of earthworms.

[CR16] Fischer E, Koszorus L (1992). Sublethal effects, accumulation capacities and elimination rates of As, Hg and Se in the manure worm*, Eisenia fetida* (Oligochaeta, Lumbricidae). Pedobiologia.

[CR17] Frazão J, de Goede RG, Capowiez Y, Pulleman MM (2019). Soil structure formation and organic matter distribution as affected by earthworm species interactions and crop residue placement. Geoderma.

[CR18] Garcia-Velasco N, Gandariasbeitia M, Irizar A, Soto M (2016). Uptake route and resulting toxicity of silver nanoparticles in *Eisenia fetida* earthworm exposed through Standard OECD Tests. Ecotoxicology.

[CR19] Giska I, van Gestel CA, Skip B, Laskowski R (2014). Toxicokinetics of metals in the earthworm *Lumbricus rubellus* exposed to natural polluted soils–relevance of laboratory tests to the field situation. Environmental Pollution.

[CR20] Hirano T, Tamae K (2011). Earthworms and soil pollutants. Sensors.

[CR142] Huang, C., Ge, Y., Yue, S., Qiao, Y., & Liu, L. (2021). Impact of soil metals on earthworm communities from the perspectives of earthworm ecotypes and metal bioaccumulation. *Journal of Hazardous Materials, 406*, 124738. 10.1016/j.jhazmat.2020.124738 10.1016/j.jhazmat.2020.124738 33316673

[CR22] ISO 11466: 1995. Soil quality—Extraction of trace elements soluble in aqua regia. International Organization for Standardization.

[CR24] ISO 19730: 2008 Soil quality—Extraction of trace elements from soil using ammonium nitrate solution. International Organization for Standardization.

[CR21] ISO 11268-1, 2, 3: 1998, 2012. Soil quality—Effects of pollutants on earthworms (*Eisenia fetida*). Part 1: Determination of acute toxicity using artificial soil substrate. Part 2. Determination of effects on reproduction. Part 3: Guidance on the determination of effects in field situations. International Organization for Standardization.

[CR23] ISO 17586: 2016. Soil quality—Extraction of trace elements using dilute nitric acid. International Organization for Standardization.

[CR25] Kabala C, Galka B, Labaz B, Anjos L, de Souza Cavassani R (2018). Towards more simple and coherent chemical criteria in a classification of anthropogenic soils: A comparison of phosphorus tests for diagnostic horizons and properties. Geoderma.

[CR26] Karaca A (2010). Biology of earthworms.

[CR27] Karczewska A, Kabała C (2017). Environmental risk assessment as a new basis for evaluation of soil contamination in Polish law. Soil Science Annual.

[CR28] Karczewska A, Krysiak A, Mokrzycka D, Jezierski P, Szopka K (2013). Arsenic distribution in soils of a former As mining area and processing. Polish Journal of Environmental Studies.

[CR29] Karczewska A, Dradrach A, Gałka B, Szopka K (2022). Does soil drying in a lab affect arsenic speciation in strongly contaminated soils?. Minerals.

[CR30] Kostecka J, Garczyńska M, Pączka G, Mazur-Pączka A (2022). Chemical composition of earthworm (*Eisenia fetida* Sav.) biomass and selected determinants for its production. Journal of Ecological Engineering..

[CR31] Kowalska J, Mazurek R, Gąsiorek M, Zaleski T (2018). Pollution indices as useful tools for the comprehensive evaluation of the degree of soil contamination–A review. Environmental Geochemistry and Health.

[CR32] Lang B, Russell DJ (2020). Effects of earthworms on bulk density: A meta-analysis. European Journal of Soil Science.

[CR423] Langdon, C. J., Piearce, T. G., Meharg, A. A., & Semple, K. T. (2003). Interactions between earthworms and arsenic in the soil environment: a review. *Environmental Pollution, 124*(3), 361–373. 10.1016/S0269-7491(03)00047-2 10.1016/s0269-7491(03)00047-2 12758017

[CR33] Langdon CJ, Winters C, Stürzenbaum SR, Morgan AJ, Charnock JM, Meharg AA, Piearce TG, Lee PH, Semple KT (2005). Ligand arsenic complexation and immunoperoxidase detection of metallothionein in the earthworm *Lumbricus rubellus* inhabiting arsenic-rich soil. Environmental Science & Technology.

[CR34] Leduc F, Whalen JK, Sunahara GI (2008). Growth and reproduction of the earthworm *Eisenia fetida* after exposure to leachate from wood preservatives. Ecotoxicology and Environmental Safety.

[CR35] Lee BT, Kim KW (2008). Arsenic accumulation and toxicity in the earthworm *Eisenia fetida* affected by chloride and phosphate. Environmental Toxicology Chemistry: an International Journal.

[CR36] Lee BT, Lee SW, Kim KR, Kim KW (2013). Bioaccumulation and the soil factors affecting the uptake of arsenic in earthworm, *Eisenia fetida*. Environmental Science and Pollution Research.

[CR422] Leveque, T., Capowiez, Y., Schreck, E., Mazzia, C., Auffan, M., Foucault, Y., Austruy, A., & Dumat, C. (2013). Assessing ecotoxicity and uptake of metals and metalloids in relation to two different earthworm species (*Eiseina hortensis* and *Lumbricus terrestris*). *Environmental Pollution, 179*, 232–241. 10.1016/j.envpol.2013.03.066 10.1016/j.envpol.2013.03.066 23688736

[CR37] Lewińska K, Karczewska A (2013). Influence of soil properties and phosphate addition on arsenic uptake from polluted soils by velvetgrass (*Holcus lanatus*). International Journal of Phytoremediation.

[CR38] Li SW, Li J, Li HB, Naidu R, Ma LQ (2015). Arsenic bioaccessibility in contaminated soils: Coupling in vitro assays with sequential and HNO_3_ extraction. Journal of Hazardous Materials.

[CR39] Liebeke M, Garcia-Perez I, Anderson CJ, Lawlor AJ, Bennett MH, Morris CA, Kille P, Svendsen C, Spurgeon DJ, Bundy JG (2013). Earthworms produce phytochelatins in response to arsenic. PLoS ONE.

[CR40] Medina-Sauza RM, Álvarez-Jiménez M, Delhal A, Reverchon F, Blouin M, Guerrero-Analco JA, Cerdán CR, Guevara R, Villain L, Barois I (2019). Earthworms building up soil microbiota, a review. Frontiers in Environmental Science.

[CR41] Müller G (1981). Die Schwermetallbelastung der sedimenten des Neckers und seiner Nebenflusse. Chemiker Zeitung.

[CR42] OECD 222. (2004). Guidelines for testing of chemicals No. 222. Earthworm reproduction test (Eisenia fetida/Eisenia andrei). OECD (Organisation for Economic Co-operation and Development), Paris.

[CR43] Papuga K, Kaszubkiewicz J, Wilczewski W, Stas M, Belowski J, Kawałko D (2018). Soil grain size analysis by the dynamometer method-a comparison to the pipette and hydrometer method. Soil Science Annual.

[CR44] Palladini J, Bagnati R, Passoni A, Davoli E, Lanno A, Terzaghi E, Falakdin P, Di Guardo A (2022). Bioaccumulation of PCBs and their hydroxy and sulfonated metabolites in earthworms: Comparing lab and field results. Environmental Pollution.

[CR45] Rahman MS, Clark MW, Yee LH, Comarmond MJ, Payne TE, Kappen P, Mokhber-Shahin L (2017). Arsenic solid-phase speciation and reversible binding in long-term contaminated soils. Chemosphere.

[CR46] Richardson JB, Görres JH, Sizmur T (2020). Synthesis of earthworm trace metal uptake and bioaccumulation data: Role of soil concentration, earthworm ecophysiology, and experimental design. Environmental Pollution.

[CR47] Rodrigues SM, Cruz N, Carvalho L, Duarte AD, Pereira E, Boim AG, Alleoni LR, Römkens PF (2018). Evaluation of a single extraction test to estimate the human oral bioaccessibility of potentially toxic elements in soils: Towards more robust risk assessment. Science of the Total Environment.

[CR48] Romero-Freire A, Peinado FJ, Ortiz MD, Van Gestel CAM (2015). Influence of soil properties on the bioaccumulation and effects of arsenic in the earthworm *Eisenia andrei*. Environmental Science Pollution Research.

[CR49] Sizmur T, Hodson ME (2009). Do earthworms impact metal mobility and availability in soil?–A review. Environmental Pollution.

[CR50] Tan K (2005). Soil sampling, preparation, and analysis.

[CR51] Wang Z, Cui Z (2016). Accumulation, biotransformation, and multi-biomarker responses after exposure to arsenic species in the earthworm *Eisenia fetida*. Toxicology Research.

[CR52] Wang Z, Cui Z, Liu L, Ma Q, Xu X (2016). Toxicological and biochemical responses of the earthworm Eisenia fetida exposed to contaminated soil: Effects of arsenic species. Chemosphere.

[CR53] Wenzel WW, Kirchbaumer N, Prohaska T, Stingeder G, Lombi E, Adriano DC (2001). Arsenic fractionation in soils using an improved sequential extraction procedure. Analytica Chimica Acta.

[CR421] Xiao, R., Ali, A., Xu, Y., Abdelrahman, H., Li, R., Lin, Y., ... & Zhang, Z. (2022). Earthworms as candidates for remediation of potentially toxic elements contaminated soils and mitigating the environmental and human health risks: A review. *Environment International, 158*, 106924, 10.1016/j.envint.2021.106924 10.1016/j.envint.2021.106924 34634621

[CR420] Yan, X., Wang, J., Zhu, L., Wang, J., Li, S., & Kim, Y. M. (2021). Oxidative stress, growth inhibition, and DNA damage in earthworms induced by the combined pollution of typical neonicotinoid insecticides and heavy metals. *Science of the Total Environment, 754*, 141873. 10.1016/j.scitotenv.2020.141873 10.1016/j.scitotenv.2020.141873 32911142

[CR54] Yang F, Xie S, Wei C, Liu J, Zhang H, Chen T, Zhang J (2018). Arsenic characteristics in the terrestrial environment in the vicinity of the Shimen realgar mine, China. Science of the Total Environment.

[CR55] Yao BM, Chen P, Zhang HM, Sun GX (2021). A predictive model for arsenic accumulation in rice grains based on bioavailable arsenic and soil characteristics. Journal of Hazardous Materials.

[CR56] Zeb A, Li S, Wu J, Lian J, Liu W, Sun Y (2020). Insights into the mechanisms underlying the remediation potential of earthworms in contaminated soil: A critical review of research progress and prospects. Science of the Total Environment.

[CR57] Zhang L, He N, Chang D, Liu X, Zhang X, Xu Y, Zhao C, Sun J, Li W, Li H, Hu F, Xu L (2018). Does ecotype matter? The influence of ecophysiology on benzo[a]pyrene and cadmium accumulation and distribution in earthworms. Soil Biology and Biochemistry.

