# Supplementary material for: Arsenic toxicity to earthworms in soils of historical As mining sites: an assessment based on various endpoints and chemical extractions
Source: Environ Geochem Health. 2023 Jun 27;45(8):6713–26. doi: 10.1007/s10653-023-01665-x (PMC10403387; doi:10.1007/s10653-023-01665-x)
Supplement: Supplementary file 1 — Supplementary file1 (DOC 100 KB) [file 10653_2023_1665_MOESM1_ESM.doc]

**Arsenic toxicity to earthworms in soils of historical As mining sites – an assessment based on various endpoints and chemical extractions**

Anna Karczewska ^1^*, Iwona Gruss ^2^, Katarzyna Szopka ^1^, Agnieszka Dradrach ^3^, Jacek Twardowski ^2^, Kamila Twardowska ^2^

^1^ Wrocław University of Environmental and Life Sciences, Institute of Soil Science, Plant Nutrition and Environmental Protection, ul. Grunwaldzka 53, PL 50-357 Wrocław, Poland

^2^ Wrocław University of Environmental and Life Sciences, Department of Plant Protection, pl. Grunwaldzki 24a, PL 50-363 Wrocław, Poland

^3^ Wrocław University of Environmental and Life Sciences, Institute of Agroecology and Plant Production, pl. Grunwaldzki 24a, PL 50-363 Wrocław, Poland

* corresponding author: Anna Karczewska, email: anna.karczewska@upwr.edu.pl

**Supplementary Materials**

**Table S1.** Soil enrichment in potentially toxic elements as assessed by a geoaccumulation index Igeo

| Soil No | Igeo ^1)^ | | | | | |
| --- | --- | --- | --- | --- | --- | --- |
|  | **As** | Cu | Pb | Zn | Ni | Cd |
| C | -1.6 | -1.2 | -1.7 | -1.4 | -1.5 | -1.3 |
| M1 | **10.1** | 2.2 | -0.4 | -0.5 | -0.2 | -1.1 |
| M2 | **9.5** | 1.4 | 2.9 | 3.0 | 0.3 | -0.1 |
| M3 | **6.9** | 0.8 | 1.4 | 0.2 | -0.6 | -1.1 |
| M4 | **5.1** | 0.3 | 0.0 | 0.3 | -0.4 | -0.7 |
| W1 | **9.5** | 3.3 | 3.3 | 1.5 | -0.9 | -0.8 |
| W2 | **8.2** | 1.6 | 1.4 | 0.7 | 0.2 | -0.3 |
|  | *Average concentrations in world soils. mg/kg (Alloway. 2013) ^2)^* | | | | | |
| *World* | *4.7* | *14* | *25* | *65* | *18* | *1.1* |

^1)^ Geoaccumulation Index (Igeo), proposed originally by Müller (1981) for river sediments, allows for the assessment of soil contamination with particular element based on its concentration in top soil horizon (C) and the value of geochemical background (GB). It is calculated according to the equation: Igeo = log_2_[C/ 1.5⋅GB], in which 1.5 is a constant introduced due to the variability of natural geological conditions. Based on the Igeo values, Müller (1981) proposed dividing river sediments into six quality classes, which was later adapted to the assessment of soil quality (Barbieri, 2016). According to this classification, the Igeo values <1 are typical for uncontaminated sediments or soils, while Igeo> 3 indicates moderately or heavily contaminated soils, and Igeo>5 denotes an extreme soil contamination.

Barbieri, M. (2016). The importance of enrichment factor (EF) and geoaccumulation index (Igeo) to evaluate the soil contamination. J Geol Geophys, 5(1), 1-4. http://dx.doi.org/10.4172/2381-8719.1000237.

Müller, G. (1981). Die Schwermetallbelastung der Sedimenten des Neckers und Seiner Nebenflusse. Chemiker – Zeitung 6, 157-164 (in German). https://doi.org/10.5026/jgeography.118.1205.

^2)^ Global geochemical background values, according to Alloway (2013).
Alloway, B.J. (Ed.). (2013). Heavy metals in soils: trace metals and metalloids in soils and their bioavailability (Vol. 22). Springer Science & Business Media. https://doi.org/10.1007/978-94-007-4470-7.

**Table S2.** Effects of soil treatment with manure or coniferous forest litter, applied at the rate 1:100 (w/w) on arsenic concentrations in soil pore water (after a 2-day incubation at 70% of water holding capacity)

| Soil No | As in soil pore water, mg/L | |
| --- | --- | --- |
|  | Untreated soil | Soil treated with manure/ or forest litter * |
| C | < 0.01 | < 0.01 |
| M1 | 5.3 | 33.0 |
| M2 | 20.5 | 80.4 |
| M3 | 7.5 | 36.3 |
| M4 | < 0.01 | 0.25 |
| W1 | 0.03 | 0.07* |
| W2 | 1.5 | 2.2 * |

* asterisks indicate the cases of soil treatment with forest litter

**Table S3.** Single correlation coefficients between the results of earthworm bioassays and the parameters of soil properties as well as the results of other bioassays (Microtox and Phytotox) – in untreated soils an soils treated with organic matter (marked with +OM). Other explanations - in the text.

| Parameter | Mass 8W | Adults | Juveniles | Cocoons | As-Eis-f | P-Eis-f |
| --- | --- | --- | --- | --- | --- | --- |
| Soil properties | | | | | | |
| As-Soil | n.s. | n.s. | n.s. | n.s. | n.s. | n.s. |
| As-HNO3 | n.s. | n.s. | n.s. | n.s. | n.s. | n.s. |
| As-AmN | n.s. | n.s. | -0,825 * | n.s. | 0,819 * | n.s. |
| As-AmN+ | n.s. | n.s. | n.s. | n.s. | n.s. | n.s. |
| As-AmN++ | n.s. | n.s. | n.s. | n.s. | n.s. | n.s. |
| pH | n.s. | n.s. | -0,814 * | n.s. | 0,821 * | n.s. |
| CEC | n.s. | -0,918 ** | n.s. | -0,903 * | n.s. | n.s. |
| P avail. | n.s. | -0,854 * | -0,826 * | -0,852 * | 0,875 * | n.s. |
| F1+F2 mg/kg | n.s. | -0,853 * | n.s. | -0,857 * | n.s. | n.s. |
| Endpoints of bioassays | | | | | | |
| Mass 8W | x | n.s. | n.s. | n.s. | n.s. | n.s. |
| Adults |  | x | n.s. | 0.999 *** | n.s. | n.s. |
| Juveniles |  |  | x | n.s. | -0.935 ** | n.s. |
| Cocoons |  |  |  | x | n.s. | n.s. |
| As-Eis-f |  |  |  |  | x | n.s. |
| Microtox | n.s. | n.s. | n.s. | n.s. | 0.827* | n.s. |
| Microtox+OM | n.s. | -0.824* | -0.911* | n.s. | 0.959** | n.s. |
| Phytotox | n.s. | -0.819* | n.s. | n.s. | 0.917** | n.s. |
| Phytotox+OM | n.s. | n.s. | -0.947** | n.s. | 0.948** | n.s. |

Asterisks stand for the significance of correlation: * p<0.05; ** p<0.01; *** p<0.001
n.s. – not significant at p<0.05
